# Optical Spectral Surveillance of Breast Tissue Landscapes for Detection of Residual Disease in Breast Tumor Margins

**DOI:** 10.1371/journal.pone.0069906

**Published:** 2013-07-26

**Authors:** J. Quincy Brown, Torre M. Bydlon, Stephanie A. Kennedy, Matthew L. Caldwell, Jennifer E. Gallagher, Marlee Junker, Lee G. Wilke, William T. Barry, Joseph Geradts, Nimmi Ramanujam

**Affiliations:** 1 Department of Biomedical Engineering, Duke University, Durham, North Carolina, United States of America; 2 Department of Surgery, Duke University Medical Center, Durham, North Carolina, United States of America; 3 Department of Biostatistics and Bioinformatics, Duke University Medical Center, Durham, North Carolina, United States of America; 4 Department of Pathology, Duke University Medical Center, Durham, North Carolina, United States of America; Tufts University, United States of America

## Abstract

We demonstrate a strategy to “sense” the micro-morphology of a breast tumor margin over a wide field of view by creating quantitative hyperspectral maps of the tissue optical properties (absorption and scattering), where each voxel can be deconstructed to provide information on the underlying histology. Information about the underlying tissue histology is encoded in the quantitative spectral information (in the visible wavelength range), and residual carcinoma is detected as a shift in the histological landscape to one with less fat and higher glandular content. To demonstrate this strategy, fully intact, fresh lumpectomy specimens (n = 88) from 70 patients were imaged intra-operatively. The ability of spectral imaging to sense changes in histology over large imaging areas was determined using inter-patient mammographic breast density (MBD) variation in cancer-free tissues as a model system. We discovered that increased MBD was associated with higher baseline β-carotene concentrations (p = 0.066) and higher scattering coefficients (p = 0.007) as measured by spectral imaging, and a trend toward decreased adipocyte size and increased adipocyte density as measured by histological examination in BMI-matched patients. The ability of spectral imaging to detect cancer intra-operatively was demonstrated when MBD-specific breast characteristics were considered. Specifically, the ratio of β-carotene concentration to the light scattering coefficient can report on the relative amount of fat to glandular density at the tissue surface to determine positive margin status, when baseline differences in these parameters between patients with low and high MBD are taken into account by the appropriate selection of threshold values. When MBD was included as a variable *a priori*, the device was estimated to have a sensitivity of 74% and a specificity of 86% in detecting close or positive margins, regardless of tumor type. Superior performance was demonstrated in high MBD tissue, a population that typically has a higher percentage of involved margins.

## Introduction

Breast cancer is an enduring health problem with more than 200,000 patients diagnosed annually in the United States [1]. Most of these patients are eligible for breast conserving surgery (BCS) [2]. BCS, also known as a partial mastectomy or lumpectomy, is a recommended treatment for early stage breast cancer and for breast cancers that have been reduced in size by neoadjuvant-therapy. The goal of BCS is to excise the tumor along with at least a 1–2 mm margin of surrounding normal tissue [3]–[5]. Post-operative histopathologic assessment of the resected specimen is the current gold standard by which completeness of excision is determined. Margin status is an important predictor of local recurrence of an invasive or *in situ* cancer after BCS [6], [7]. Unfortunately, as many as 17.7–72% of patients undergoing BCS require repeat surgeries due to a close or positive surgical margin [3], [4], [8]–[12]. One recent study observed that in over 2,000 women undergoing BCS, the variation in re-excision rate varied from 0–70% across surgeons, indicating that there is no reliable intra-operative standard for preventing re-excision [13]. Younger women in particular tend to have higher percentages of involved margins and higher local recurrence rates [6], [14]–[19]. These age-dependent findings may be due to increased breast density; a study by Bani et al [20] found that higher mammographic breast density (MBD) was associated with higher re-excision rates, 18% (MBD-1), 18% (MBD-2), 22% (MBD-3), and 42% (MBD-4). In the U.S., touch-prep cytology and frozen section analysis have been used to help address this need intra-operatively. However, these techniques require a trained pathologist to be present, prolong surgery time (20–40 minutes), and have technical challenges associated with processing fatty breast tissues. By 2015, it is expected that the number of patients undergoing BCS will increase from approximately 200,000 to more than 270,000 per year in the U.S., at an annual growth rate of 5.5% [2].

The best available method for the detection of residual carcinoma on a surgical tumor resection specimen is post-operative histopathology, which is the gold standard. This approach uses light microscopy to detect the presence of disease in 4–5 micrometer-thick tissue sections (at micrometer image resolution), taken from ∼3 mm thick slices of the tumor margin (millimeter sampling frequency). An ideal intra-operative tool would sample tumor margins at comparable or better sampling frequency and image resolution. However, the need for microscopic resolution results in a practical sampling limitation, since there is inherent difficulty in sampling, imaging and analyzing large tissue areas (ca. 10–100 cm^2^) with microscopic resolution in intra-operative time frames [21]. This is a particular problem in heterogeneous organs such as the breast, in which samples are routinely large, and it is not possible to grossly observe and preferentially sample small areas of residual disease, due to the surrounding mix of normal tissue types including fat, glands, and fibrous tissues. Achieving microscopic resolution of the tumor margin in an intra-operative tool also comes at the expense of sensing depth. In an effort to address this important problem, our group has developed a strategy to quantify the morphological features of the breast tumor margin over a wide field of view by creating quantitative hyperspectral maps of the tissue optical properties (absorption and scattering) where each voxel can be deconstructed to provide information on the underlying tissue composition [21]–[25]. This strategy provides a means to quickly survey centimeter-square tissue areas with quantitative analysis of spectral information serving to provide a surrogate for microscopic imaging resolution. Further, the visible spectral range (450–600 nm) provides the requisite sensing depth of 2 mm [22], a frequently used cut-off for negative margins. The primary absorbers in the breast over this wavelength range include β-carotene stored in adipocytes (reflective of fatty tissues) and hemoglobin found in blood cells in the vasculature (reflective of tissue vascularity). Likewise, the scattering properties are directly related to the collagen and cell density within the breast (reflective of fibroglandular content).

**Figure 1:**
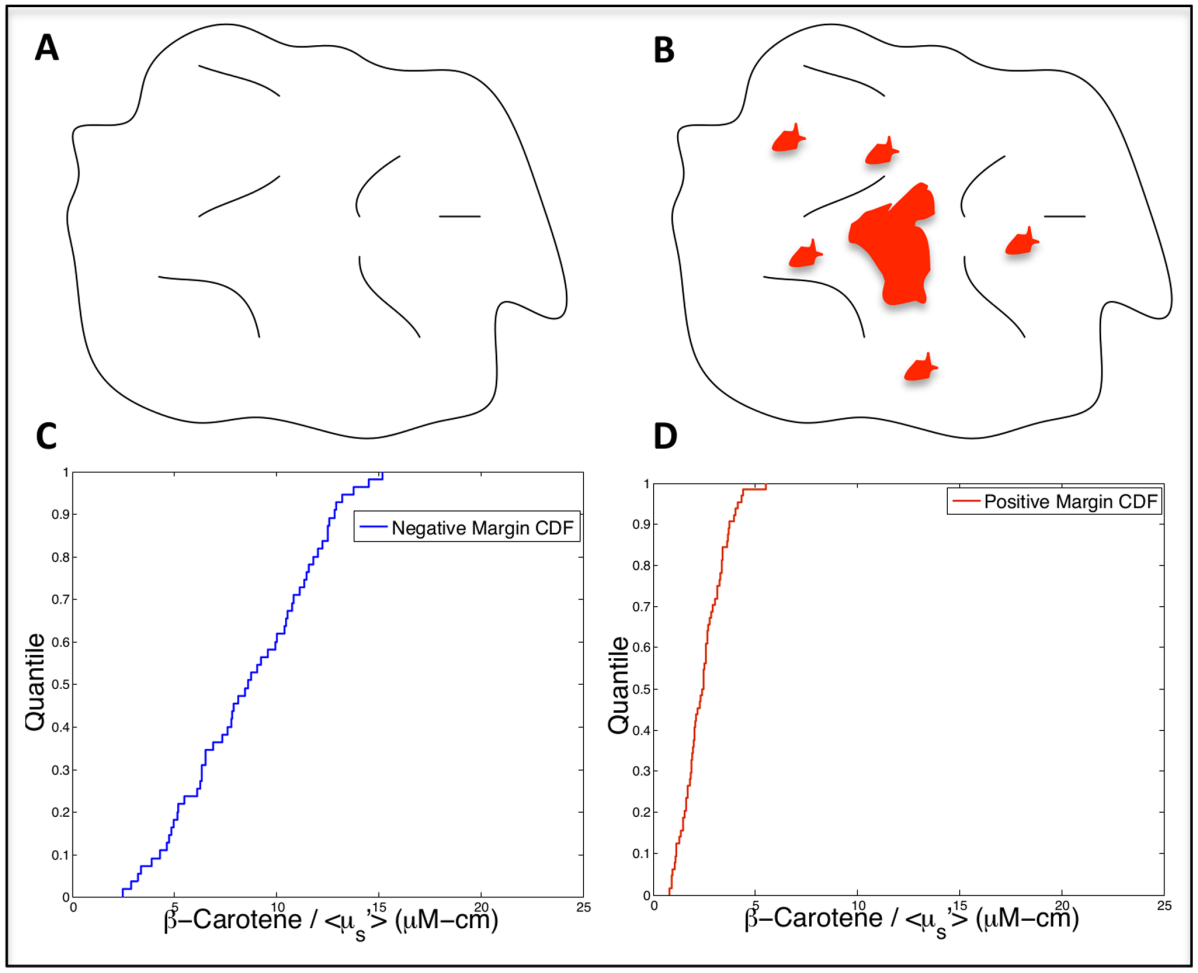
Schematic of our conceptual approach. Cartoon representing A) a “negative” tumor margin surface corresponding to a mix of normal tissues, and B) a “positive” tumor margin surface with areas of residual tumor (red) at the surface. The cumulative distribution functions in C) and D) are actual optical data from representative margins (sensitive to the relative amount of fat and fibroglandular tissue). In a negative margin (C), there is a distribution of values, which corresponds to the mixture of tissue types in benign (aka negative) margins. When malignancy is present in varying amounts (D), a shift in the optical contrast distribution is observed, due to the disruption in the tissue landscape caused by the increase in cancerous tissue and displacement of normal tissue.

The challenge for any intra-operative technique for breast tumor margin assessment is the ability to detect the *signal* (i.e., the histologic changes due to varying amounts of malignancy at the margin) over the *noise* (i.e., the normal inter-patient and intra-patient variation in breast composition). If we view the range of normal tissues in the breast as a “landscape,” then the challenge in margin assessment is to detect the presence of malignant tissue at the boundary of an otherwise “normal” margin, as a perturbation in that landscape. Our hypothesis was that quantitative diffuse reflectance mapping would be sensitive to those shifts, by leveraging the interactions of light with tissue at the molecular and histological level to sense the tissue composition (our conceptual approach is outlined in Figure 1). To demonstrate that the technology could meet this task, we first established that the information inherent in spectral data was specifically related to salient tissue composition and micro-morphologic features in the breast. Specifically, the relationship between the quantitative metrics obtained from the absorption and scattering endpoints, and the proportion of fat and collagen/glands-quantified from histopathology of specific sites from negative and positive margins were quantified. Next, the ability of this spectral mapping technique to survey shifts in of the morphological features of the normal breast were determined, by analyzing the spectral information arising from inter-patient variations in mammographic breast density (MBD), which further established the morphological features to which the hyperspectral maps are sensitive. Finally, the utility of this surveillance approach to detect shifts in the histologic landscapes caused by the presence of residual carcinoma was assessed by imaging breast tumor resection margins intra-operatively in 70 patients undergoing BCS, and predicting the presence of residual disease through a statistical predictive modeling approach for automated, unbiased selection of predictor variables.

## Materials and Methods

### Ethics Statement

This study was performed in strict accordance with a protocol approved by the Duke University Institutional Review Board. Patients over age 18 undergoing BCS granted written consent under the approved clinical protocol.

### Patient Population

The following characteristics were recorded for each patient (if available): radiographic breast density, menopausal status, neoadjuvant treatment status (chemotherapy or endocrine therapy), age, body mass index (BMI), and surgical re-excision status. For the analyses in this manuscript, data was only included from patients who had not undergone prior radiation, adjuvant treatment, or surgery 1) due to limited sample sizes and 2) in order to assess differences in surgical margin status without these additional confounding factors. For mammographic breast density (MBD), each patient was assigned a value based on their pre-surgery mammogram: 1 (fatty), 2 (scattered fibrous), 3 (heterogeneously dense), or 4 (extremely dense). For the analyses in this paper an MBD score of 1 or 2 was considered to be low density, while a score of 3 or 4 was considered to be high density; the data was binned this way since the majority of the patients had 2′s or 3′s.

### Instrumentation

The Quantitative Diffuse Reflectance Imaging (QDRI) instrument has been described in detail in prior publications [21]–[23], [26], [27], and consists of a Xenon lamp coupled to a monochromator (Gemini 180, Jobin-Yvon HORIBA), an 8-channel fiber-optic imaging probe, an imaging spectrograph (Triax 320, Jobin-Yvon HORIBA), and a 2-D CCD camera (Symphony, Jobin-Yvon HORIBA). This system can be used to measure diffuse reflectance spectra from 8 discrete sites in a single acquisition. For ease of use and to avoid crosstalk between adjacent probes at the tissue surface, the 8 channels of the probe were secured in an aluminum adaptor in a 4×2 array with a center-to-center separation of 10 mm between each channel; a 5 mm sampling resolution was achieved by translating the probe over the tissue in 5 mm increments. This sampling resolution is comparable to the sampling resolution of pathology where tissue sections are bread-loafed and examined in ∼3 mm intervals. The sensing depth of the probe was previously simulated to be 0.5–2.2 mm and is tissue dependent with the deepest penetration depth in adipose tissues [22]. This analysis was carried out retrospectively with a Monte Carlo model, which incorporated the optical properties of adipose, fibroglandular, and malignant breast tissue obtained from our clinical study. A custom software application was written in-house using LabVIEW 8.5 (National Instruments, Austin, TX) and MATLAB R2008a, and was used for instrument control and data acquisition and processing. Prior to imaging, the system was calibrated for optical throughput with a one-time measurement of a Spectralon diffuse reflectance standard (LabSphere, North Sutton, NH).

### Measurement Procedure

Partial mastectomy specimens were excised and oriented by the surgeon using surgical clips and sutures to mark the center of 4 of the 6 total margins. Specimens then underwent routine specimen mammography. QDRI imaging of the excised lumpectomy specimen was performed intra-operatively, either in the operating room or in an adjacent room. Approximately 16±5 minutes post-excision, the specimen was placed in a rectangular plexi-glass box for imaging, oriented such that the clip/suture was at the center of the box face. The imaging probe was interfaced to the lumpectomy specimen via holes in the plexi-glass box. Diffuse reflectance measurements (450–600 nm) were collected from the tissue surface with 5 mm sampling resolution until the entire margin had been measured by simply translating the imaging probe to sample the interleaving pixels [21]–[23]. Four surgeons participated in this study. The surgeon was blinded to the imaging output and performed selective intra-operative re-excision based on only on the review of the specimen mammograms and gross examination, as is standard of care. Data collection, processing, and display of optical images were fully automated in the QDRI imaging platform. Once the specimen was placed in the plexi-glass box, acquisition-time of spectra from a single probe placement (8 spectra simultaneously) varied on the order of several hundred milliseconds, and the analysis time was 0.5 seconds per spectrum. Therefore, the time to acquire and display the results from a single probe placement was on the order of 5–10 seconds. In this study, the time required to image a single resection margin (20–40 cm^2^) was typically 20–25 minutes, which included loading the specimen, collecting the data, book-keeping, inking for pathologic co-registration, and data analysis. However, the time required for just translating the probe, acquiring the data, and processing the images was on the order of 5–10 minutes per margin. (The bottle-neck in this process was translating the probe; a newer version of the technology decreases this bottle-neck by increasing the number of parallel channels to 49).

### Margin Level Histology

Following imaging of the full margin surface, the four corners of the measured margin were marked with histological ink for later pathologic correlation with the imaged area (referred to as margin-level analysis). The orientation of the imaged margin was recorded and sent to surgical pathology to ensure orientation concordance. The specimen was further inked by surgical pathology per standard protocol. Post-operative pathology served as the standard to classify each margin as negative (malignant cells >2 mm from tissue surface), close (malignant cells ≤2 mm from tissue surface), or positive (malignant cells at surface). For the purposes of this study, the close and positive margins were lumped together as “positive”.

### Site Level H&E Image Analysis

In addition to the margin-level analysis, up to 10 sites per margin were inked with a unique color histological ink at the time of optical assessment and denoted as “research sites,” which were analyzed in detail to provide a histologic assessment of the underlying tissue composition; this site-level analysis is described in greater detail by Kennedy et al [27]. In the cases where individual imaged pixels were marked with ink, the resulting pathologic tissue sections could be paired with quantitative optical parameters from those pixels. In the work by Kennedy et al [27], extracted optical parameters for each research site were paired with the overall diagnosis (adipose, fibroadipose, fibroglandular, fibrous, malignant, etc.), and Wilcoxon rank-sum testing was used to detect significant differences in optical parameters between diagnostic categories. To further investigate the relationship between optically-measured β-carotene concentration, reduced scattering coefficient, mammographic breast density, and tissue micromorphology in adipose tissues specifically, hematoxylin and eosin (H&E) stained sections of histologically confirmed adipose tissues (n = 84) were digitally imaged. 53 tissue sections from 16 patients in the low MBD subgroup, and 31 tissue sections from 9 patients in the high MBD subgroup were digitally imaged (mean of 3.4 and 3.3 sections per patient, respectively). Patients were further matched for body-mass index (BMI) in the range of 25–30, which left 9 sections from 2 high MBD patients and 16 sections from 3 low MBD patients. The images were acquired with a Zeiss Axio Imager upright microscope with a 10% neutral density filter, 10X objective, a halogen light source, and a QImaging MicroPublisher 5.0 MP color camera. MetaMorph 7.6.5 was used to adjust the acquisition time and RGB gain. The field of view (FOV) for the adipose images was 1 mm×1.3 mm with a resolution of 1.1 µm.

Digital images of the H&E-stained adipose tissue sections were analyzed with an automated image processing algorithm to extract average cell area and cell density. The green channel of the RGB images obtained from the color camera was used in the algorithm, as it provided a convenient method of separating the primarily pink and blue stained tissue from white fat. All images were preprocessed with a 2-D implementation of an edge-preserving bilateral filter. Subsequently, the MATLAB implementation of the Canny edge detector was used to extract the outlines of the adipocytes. The interior of each outlined shape was measured and the number of shapes was counted to provide an estimate of cell density. Empirically determined cell-area thresholds of 129.3 µm^2^ and 22,569 µm^2^ were used to limit the counted results to those with a high probability of being an adipocyte.

### Spectral Data Analysis

Details of the analysis of the diffuse reflectance data from the partial mastectomy specimens can be found in prior publications. For each site, diffuse reflectance spectra were calibrated using a Spectralon 99% reflectance standard (Labsphere, North Sutton, NH). Collection of throughput-calibrated diffuse reflectance spectra from each site on the surface of each specimen allowed creation of a spectral reflectance cube *R(x,y,λ)*, where *R* is calibrated reflectance, *x* and *y* are spatial coordinates of the tissue surface, and *λ* is wavelength. An inverse Monte Carlo model [24], [25] was then used to extract the wavelength-dependent absorption and reduced scattering coefficients from the diffuse reflectance spectra from each site (or pixel) on the spectral image. Diffuse reflectance spectra measured from the tissue were fit over the wavelength range of 450–600 nm. The free parameters related to absorption were the concentrations of the intrinsic absorbers in this wavelength range, namely, oxygenated hemoglobin (HbO_2_), de-oxygenated hemoglobin (Hb), and [β-carotene]. Lymphazurin™ (Tyco Healthcare), a blue dye injected peri-tumorally prior to surgery to locate the sentinel node during surgery, was also included as an extrinsic absorber since it was present in many of the samples. For scattering, the fixed parameters were the refractive indices of the scatterers (1.4) and the surrounding medium (1.36), and the anisotropy factor (0.8), whereas the free parameters were the size and density of the spherical scatterers.

Taking these concentrations directly, the spectral absorption coefficient cube µ*_a_(x,y,λ)* was reduced to a series of 2-D surface maps *A_n_(x,y)*, which are the maps of the *n^th^* absorber *A* at each (x,y) location. Likewise, the reduced scattering coefficient cube was reduced to *<*µ*_s_′>(x,y),* where *<*µ*_s_′>* is the wavelength-averaged reduced scattering coefficient. This allowed the creation of surface parameter maps of the tissue, which reflects either absorber concentrations (oxyhemoglobin, deoxyhemoglobin, total hemoglobin, β-carotene, or Lymphazurin™) or a summary of the scattering characteristics of the tissue, or combinations thereof.

### Empirical Cumulative Distribution Functions

Using all pixels from all parameter maps acquired from lumpectomy specimens, four empirical cumulative distribution functions (eCDFs) for each parameter were created for positive and negative margins from high density and low density breasts (negative, high density; negative, low density; positive, low density; positive, high density). To calculate statistical differences between the eCDFs, empirical p-values for a Kolmogorov-Smirnov statistic were computed using blocked permutation to maintain the correlation structure of multiple site level measurements within each margin.

### Image Reduction

Three approaches were adopted for reducing the 2-D parameter maps to image descriptive scalar variables, which are more easily paired with the overall binary margin diagnosis. The first approach was to simply take the median value of the image. The second approach was to quantify differences in the distribution of values within an image by computing the percentage of image pixels, which lie below a particular value threshold for each image. For each of the 5 quantitative parameter maps (β-carotene, total hemoglobin, <µ_s_′>, β-carotene/<µ_s_′>, and total hemoglobin/<µ_s_′>), 19 variables were computed which represented 19 thresholds. These thresholds were pre-selected for each quantitative parameter by placing all image pixels from all margins into a single vector, and then selecting the 0.05–0.95 data quantiles in 0.05 quantile increments as the threshold values. This method ensured that the threshold values were selected to evenly split the data on the actual data distribution, as opposed to the data range (which is sensitive to outliers and extremes in the data). Then, for each individual margin sample and quantitative parameter combination, these 19 thresholds were used to compute the percentage of image pixels below each threshold. The third and final approach was a statistical approach which, compared the distribution of a given image’s pixels to the distribution of all pixels from all (positive + close) margins. Specifically, the two-sided Kolmogorov-Smirnov test was used to compute the likelihood that the distribution of pixels in the image of interest came from a “positive” pixel distribution, and the resulting Kolmogorov-Smirnov statistic was directly used as an image-descriptive variable.

Based on the image-reduction schemes outlined above, for each margin, a set of 105 image descriptive values were computed (5 quantitative optical parameters×(19 thresholds +1 image median +1 Kolmogorov-Smirnov statistic) = (5×21) = 105 total variables). For each quantitative optical parameter, a single threshold value which best separated positive/close margin samples from negative margin samples was determined by calculating Wilcoxon rank sums and selecting the threshold value with the lowest *p*-value. Thus, a final set of 5 quantitative optical parameters× (1 optimum threshold +1 image median +1 Kolmogorov-Smirnov statistic) = (5×3) = 15 image descriptive variables per imaged margin were available for construction of a predictive model.

### Conditional Inference Tree Models

A conditional inference tree (CIT) model for automated selection of predictor variables and estimation of prediction accuracy was employed [28]. This was accomplished using the ‘ctree’ function of the library (party) in the R programming environment. All 15 candidate variables for all imaged margins were available for selection by the conditional inference tree (CIT) model. The CIT model-building process resulted in the selection of a subset of the predictor variables, and optimum cut-points on those variables, which gave the best classification accuracy for the entire patient cohort. The statistical significance of the models (including the entire model-building or variable selection aspect of the models) was computed using Fisher’s exact test (1000 permutations), by randomly sampling the margin diagnosis vector without replacement to shuffle the margin classifications, before building the CIT models. For each of the permutations, the performance of the permuted model was compared against the performance of the observed (true) model using the quantity *F = (1-sensitivity)^2^+(1-specificity)^2^,* which is minimized in well-performing models. The *P*-value (probability of a random model performing better than the observed model) was computed by determining the fraction of *F_permuted_*<*F_observed_*.

## Results and Discussion

Table 1 contains a breakdown of statistics for patients enrolled stratified by mammographic breast density. Note that in the subsequent results, all margin statistics are related to the margin status of the initial excised surgical specimen only, and do not include information about intra-operative re-excision specimens; therefore, the positive margin rates reported here are not the same as the final margin status for these patients (since the surgeons in some cases performed an intra-operative re-excision which “corrected” a positive or close margin on the primary specimen). In addition, a significant number of imaged margins were those from the anterior and posterior positions on the specimen. In cases where a close or positive margin was identified in these locations, if the surgeon had removed associated anterior skin or the posterior pectoral fascia, no additional surgery or re-excision would have been anticipated in these patients. Margins were imaged in 99 neoadjuvant-therapy naïve patients; 88 margins imaged from 70 patients were retained for analysis in this paper. Patients were excluded due to instrumentation failure (n = 3), discrepant pathological orientation (imperfect co-registration between the imaged area and the margin surface as delineated by pathology, n = 20), atypical ductal hyperplasia (n = 1), re-excision lumpectomy (n = 2), and incomplete images (n = 3). The low density group consisted of 11 MBD-1 patients and 27 MBD-2 patients; the high density group consisted of 25 MBD-3 patients and 7 MBD-4 patients. No significant differences (using a Wilcoxon rank-sum) were observed between breast density subgroups for invasive tumor component size (p = 0.76), *in-situ* tumor component size (p = 0.77), or lumpectomy volume (p = 0.1). There was a borderline significant result for age (p = 0.06), reflecting the fact that breast density can be decreased with age although there is significant variability between patients.

**Table 1:** Patient and tumor demographics.

	Low MBD Patients	High MBD Patients
**# of Patients**	38	32
**Avg. Age (range)**	61.9 (43–87)	56.8 (36–83)
**Avg. BMI (range)**	31.7 (18.3–49.2)	28.1 (18.4–43.7)
**Tumor Receptor Status**		
ER +,−	33 (86.8%), 5 (13.2%)	27 (84.4%), 4 (12.5%)
PR +,−	29 (76.3%), 9 (23.7%)	25 (78.1%), 6 (18.8%)
HER-2/neu +,−	0 (0%), 33 (86.8%)	5 (15.6%), 20 (62.5%)
Triple-negative	4 (10.5%)	0 (0%)
**Avg. Lumpectomy Volume (range)**	63.7 cm^3^ (10.2–192.0 cm^3^ )	49.5 cm^3^ (9.5–175.9 cm^3^ )
**Primary Tumor Histology**		
IDC	4 (10.5%)	3 (9.4%)
DCIS	4 (10.5%)	5 (15.6%)
IDC/DCIS	16 (42.1%)	17 (53.1%)
Other	4 (10.5%)	3 (9.4%)
No Tumor	0 (0%)	1 (3.1%)
**Avg. Invasive Component Size (range)**	1.68 cm (0.6–4.3 cm)	1.59 cm (0.4–3.6 cm)
**Avg. In situ Component Size (range)**	3.33 cm (0.1–10 cm)	3.04 cm (0.1–7 cm)
**Measured Margin Histology**		
IDC	8 (16.7%)	6 (15.0%)
DCIS	8 (16.7%)	9 (22.5%)
IDC/DCIS	1 (2.1%)	1 (2.5%)
Other	6 (12.5%)	7 (17.5%)
No Tumor	25 (52.1%)	17 (42.5%)
**Surgical Margin Status**		
Negative (>2 mm)	25 (52.1%)	17 (42.5%)
Close (<2 mm)	15 (31.3%)	14 (35.0%)
Positive	8 (16.7%)	9 (22.5%)
**Measured Margin**		
Anterior	10 (20.8%)	7 (17.5%)
Posterior	11 (22.9%)	12 (30.0%)
Superior	7 (14.6%)	8 (20.0%)
Inferior	8 (16.7%)	4 (10.0%)
Medial	6 (12.5%)	6 (15.0%)
Lateral	6 (12.5%)	3 (7.5%)

BMI – body mass index, IDC – invasive ductal carcinoma, DCIS – ductal carcinoma *in situ*.

### Spectral Contrast is Sensitive to Differences in Benign Breast Composition

With quantitative spectral imaging, the concentrations of β-carotene and hemoglobin can be quantified, which should be directly related to the amount of fatty tissue and vasculature, respectively. Likewise, the reduced scattering coefficient has been shown to be sensitive to changes in cellular density and collagen content [29]. We chose to exclude hemoglobin from our analysis because of a study we performed on the post-excisional kinetics of that parameter in freshly bisected mastectomies and fresh lumpectomy specimens using our device, in which we found that hemoglobin is not a stable diagnostic parameter post-excision [26]. Since our quantitative technique explicitly determines the molar concentration of each relevant absorber in the spectral range, no further correction is needed to account for the effects of hemoglobin on our measurements. In addition, shielding of β-carotene absorption by hemoglobin is not a concern due to the fact that β-carotene absorbs primarily in the spectral window between the Soret band and alpha and beta absorption bands where hemoglobin absorption is low. Therefore, for the purposes of this study, the hemoglobin content was not considered further.

**Figure 2:**
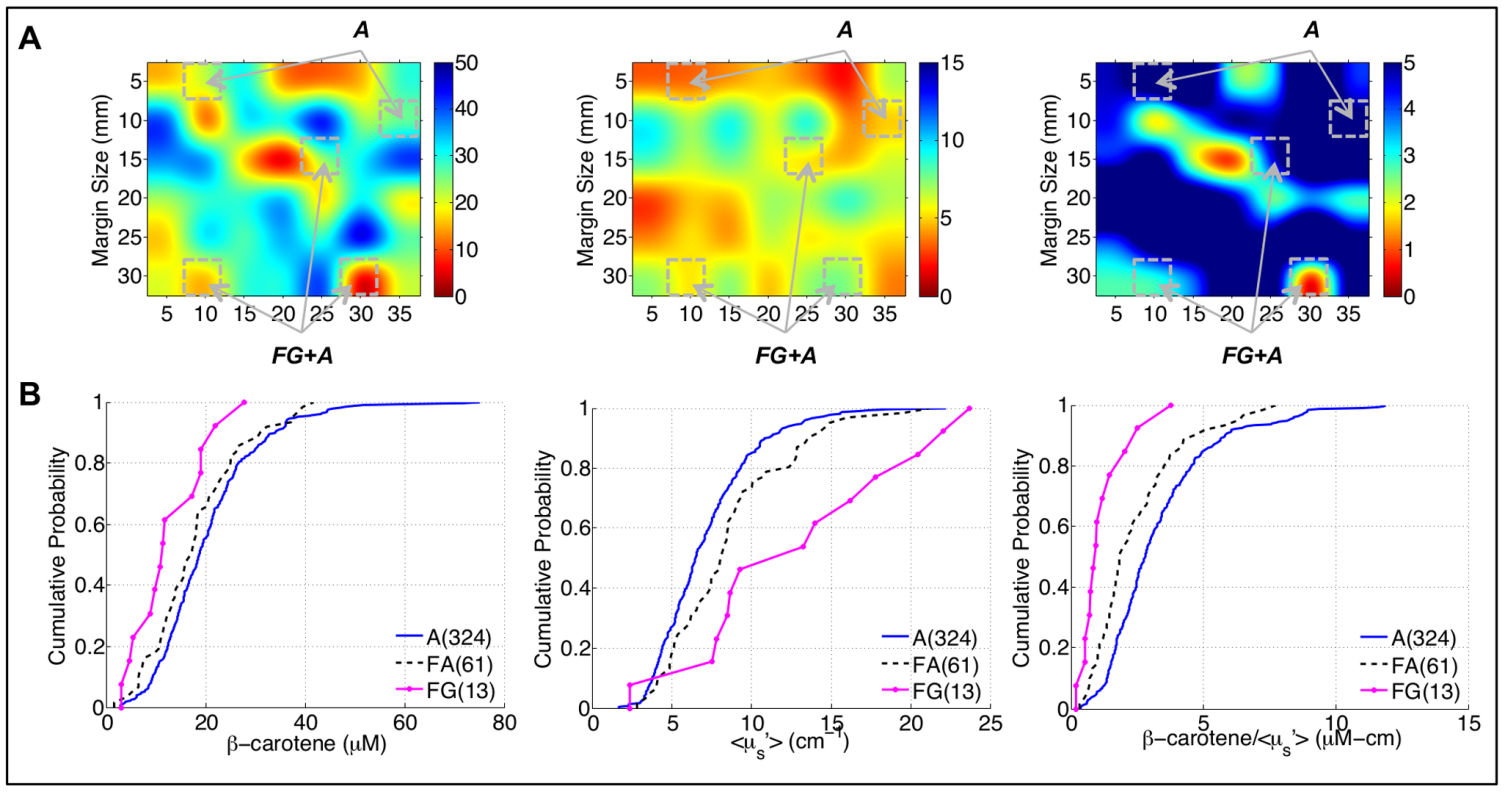
Relationship between optical parameters and benign breast composition. A) 50x bicubic interpolated images of [β-carotene], <µ_s_′>, and [β-carotene]/<µ_s_′> from a negative margin. Sites with corresponding histopathology are highlighted with diagnoses of adipose (A) or fibroglandular plus adipose (FG+A). B) Empirical cumulative distribution functions (eCDFs) of the site-level data for fibroglandular (FG), fibroadipose (FA), and adipose (A) sites.

It was previously hypothesized that [β-carotene]/<µ_s_′> is sensitive to the relative amount of fat (from the [β-carotene] parameter) to fibroglandular tissue (from the <µ_s_′>parameter) [23]. Figure 2A contains an image of [β-carotene], <µ_s_′>, and [β-carotene]/<µ_s_′> from a negative margin with histologically-confirmed adipose, and fibroglandular plus adipose, sites highlighted. The sites with both fibroglandular and adipose tissue have lower [β-carotene] and higher <µ_s_′>, and lower [β-carotene]/<µ_s_′> compared to purely adipose tissue as expected. These trends were confirmed in a larger cohort of histologically-confirmed sites from the margins imaged in this study where H&E images of the sites were broken down into specific categories: fibroglandular (collagen and benign epithelium), fibroadipose (collagen and adipose), and adipose. The empirical cumulative distribution functions (eCDFs) in Figure 2B show that [β-carotene] decreases, <µ_s_′> increases and [β-carotene]/<µ_s_′> decreases as the tissue changes from predominantly adipose tissue to predominantly fibroglandular tissue components (i.e. decreasing fat and increasing collagen/glands).

### Spectral Surveillance Tracks Shifts in Tissue Composition Related to Breast Density

**Figure 3:**
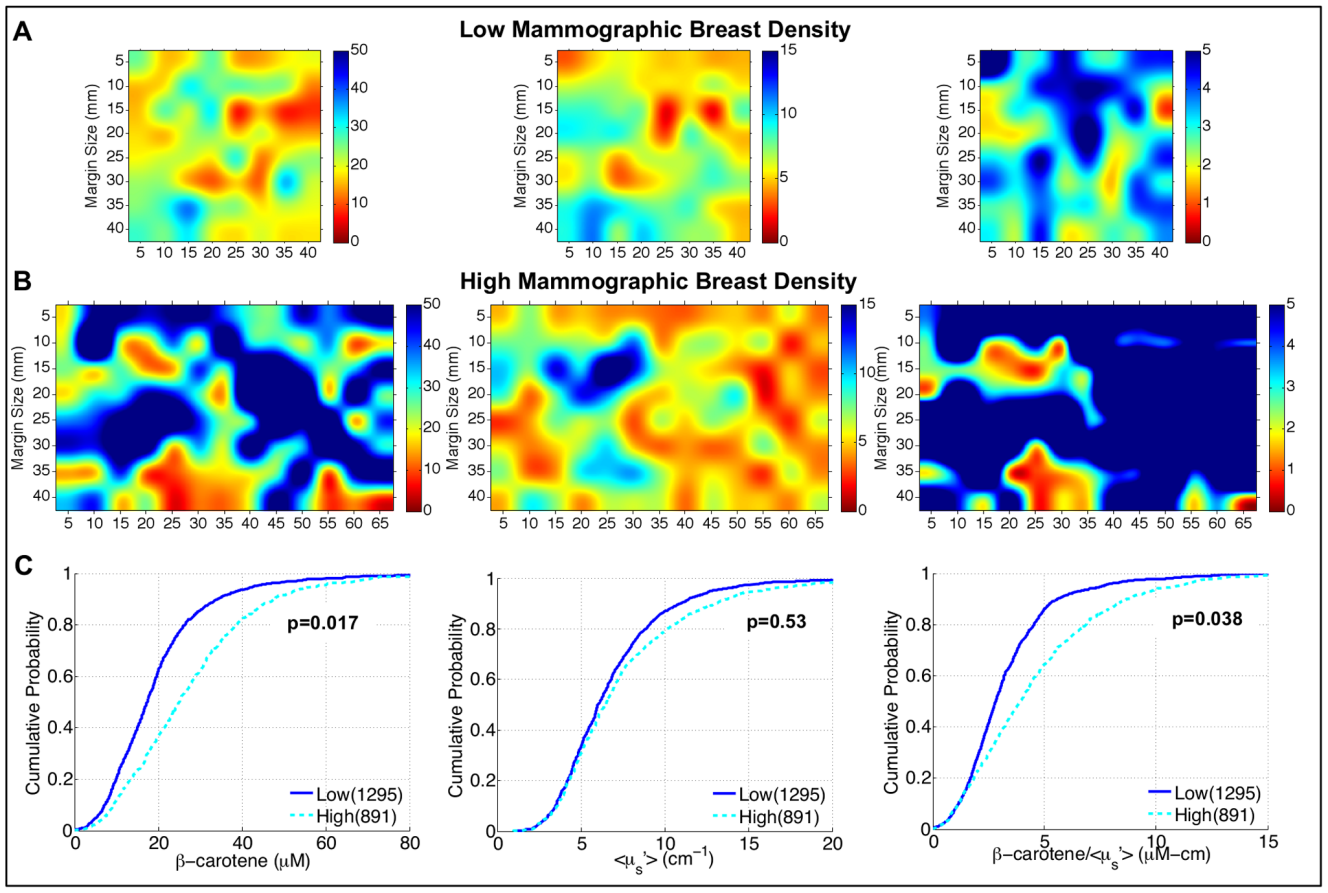
Relationship between optical parameters and benign breast composition with differences in breast density. A) Parameter maps from a low density margin; B) Parameter maps from a high density margin; blue indicates higher values of the corresponding variable. C) eCDFs of all measured sites from negative, neoadjuvant-naïve margins, separated by mammographic breast density. *P*-values were calculated with modified Kolmogorov-Smirnov statistics.

To further elucidate the sensitivity of this technique to the histological landscape in the breast when it is used to “map” large areas, mammographic breast density (MBD) in negative margins was used as a model system. It is expected that as breast density increases, the percentage of collagen/glands in the overall breast should increase whereas the percentage of fat should decrease. Based on the findings in Figure 2 this would suggest a decrease in [β-carotene] and an increase in scattering. To test this hypothesis, the spectral information from negative neoadjuvant-naïve margin images was used to investigate whether the β-carotene and <µ_s_′>parameters would reflect this shift due to differences in breast density between patients. Figure 3 shows a representative set of images and eCDFs of all pixels from negative margins, separated by low (MBD = 1 or 2) and high (MBD = 3 or 4) density breasts. Interestingly, [β-carotene] and [β-carotene]/<µ_s_′> are significantly higher in the negative margins of high density compared to low density breasts, which is counter intuitive (since low density breasts are associated with higher proportions of fatty tissues). The increased <µ_s_′> is likely associated with an increase in glandular and collagen tissue in the breasts of these patients. However, the significant increase in [β-carotene] with breast density, and thus the ratios of [β-carotene] to <µ_s_′>, could not be attributed to differences in the relative percentage of adipose tissue, since low density breasts should by definition have a higher percentage of this tissue type. This result suggested that the adipocytes in high density breasts have a higher baseline β-carotene concentration than those in low density breasts.

### Adipocytes in High Density Breasts are Smaller and have a Higher Baseline β-carotene Concentration

Under normal conditions β-carotene is absorbed through the intestines and circulates in the blood before being transported to the liver; excess β-carotene is stored in adipose tissue [30]–[32]. Inside the adipocyte, β-carotene and retinol (a byproduct of β-carotene) are converted into retinaldehyde; retinaldeyde can then be converted into retinoic acid [33]. This β-carotene/retinoic acid pathway is important in adipocyte differentiation and has also been shown to play an important role in modulating adipocyte size [33], [34]. This suggests that β-carotene concentration is related to adipocyte size, which prompted an investigation into whether differences in adipocyte size existed between high and low density breasts.

**Figure 4:**
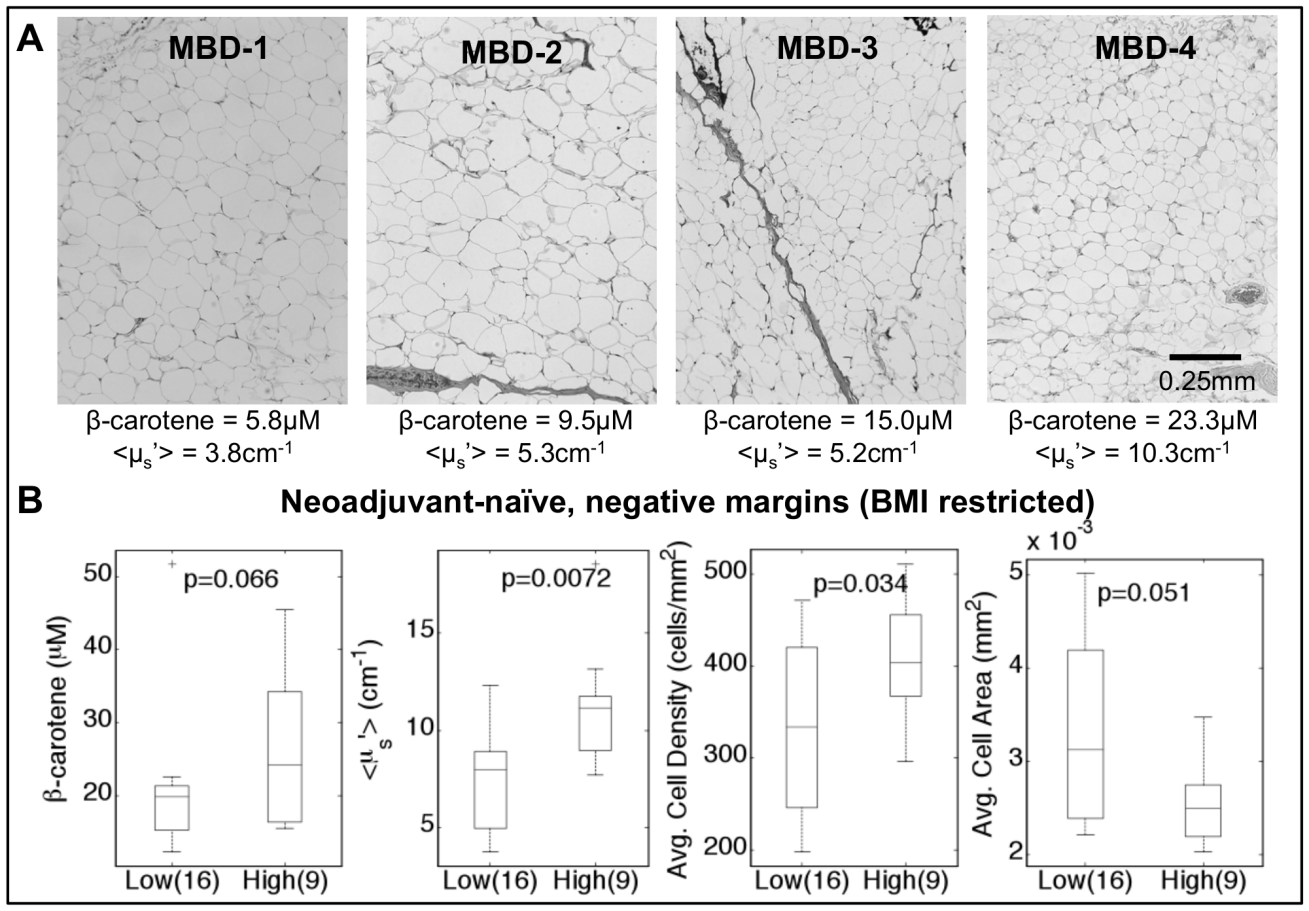
Analysis of adipose tissue between low and high density breast tissue. A) Representative H&E micrographs (100x) from all 4 mammographic breast densities (MBD). Cell area and cell density were calculated from an automated image analysis algorithm applied to H&E slides. [β-carotene] and <µ_s_′> were measured via quantitative spectral imaging. B) The adipose sites are from the negative margins of neoadjuvant-naïve patients with a BMI restricted to 25–30. P-values were calculated with a Wilcoxon rank-sum.

Digital H&E images of optically-sampled adipose tissues from breasts covering the full range of density classes are shown in Figure 4A. Qualitatively, the average adipocyte size appears to decrease as breast density increases. We compared the adipocyte morphological metrics computed from digital images of tissue sections; an average of 3.4 and 3.3 tissue sections per patient were imaged for the low and high MBD subgroups, respectively, in order to sample the intra-patient variability. A quantitative analysis of the adipose tissue images (n = 84) showed increased adipocyte density (p = 0.093) and smaller adipocyte areas (p = 0.17) in the adipose tissues of high density breasts (MBD-3 and MBD-4) as compared to low density breasts (MBD-1 and MBD-2). After restricting the body mass index (BMI) range of the patients included in this analysis to BMI = 25–30 (n = 25) to correct for the influence of BMI on adipocyte size, statistically significant differences were observed: namely, increased adipocyte density (p = 0.034) and smaller adipocyte areas (p = 0.05) in the adipose tissues of high density breasts as compared to low density breasts (Figure 4C). This BMI range was chosen since the majority of the patients fell within this range. This analysis suggests that increased [β-carotene] is associated with smaller adipocytes, and that high density breasts overall have smaller adipocytes, thus resulting in an increased baseline level of [β-carotene] in the fatty tissues of patients with high breast density. Adipose tissues from high density breasts had higher scattering values and smaller adipocytes, indicating larger scattering signals from smaller cells that are more tightly packed.

### Spectral Surveillance of Shifts in Breast Tissue Composition can be Leveraged for Detection of Residual Cancer on a Tumor Margin

**Figure 5:**
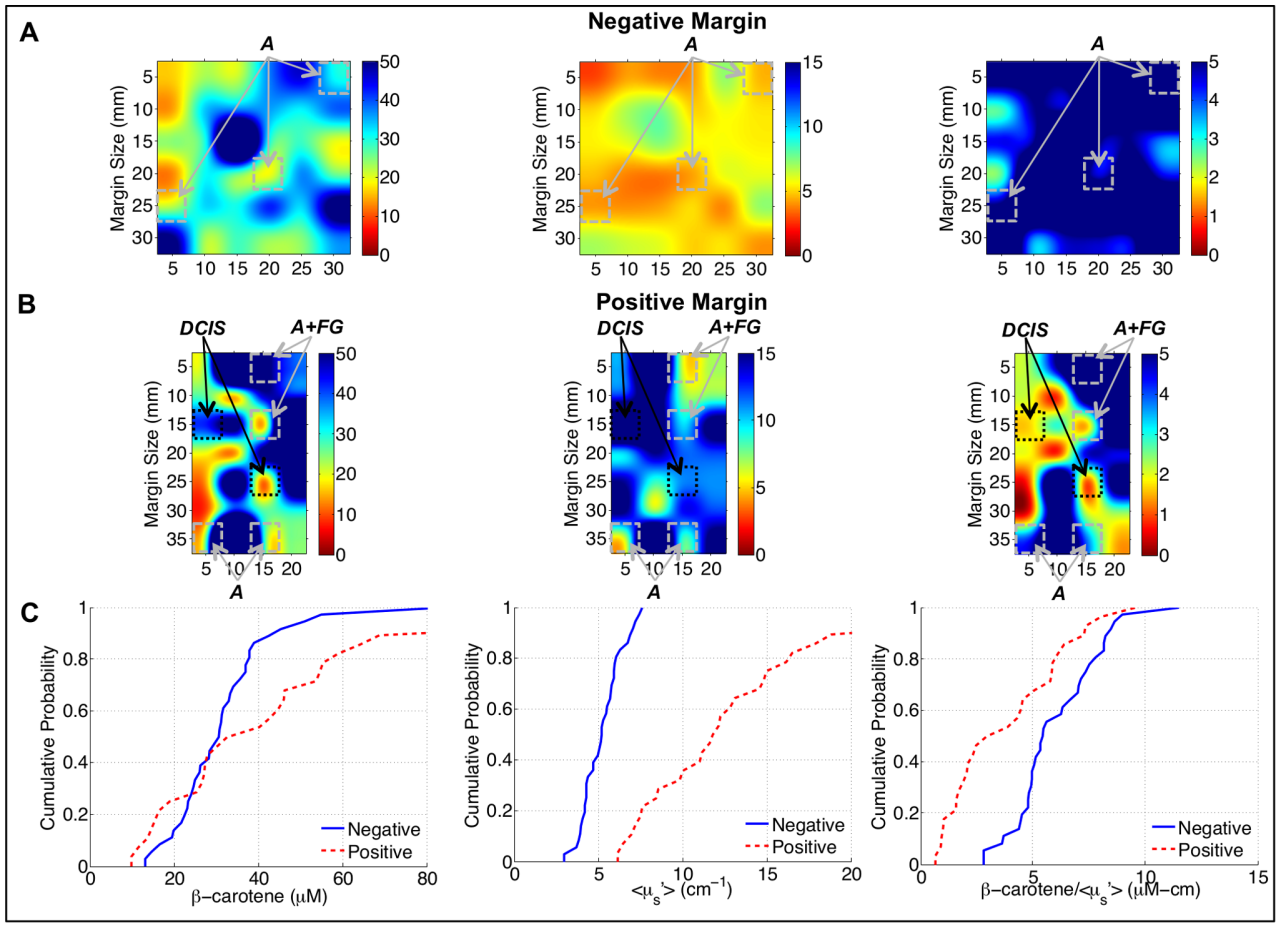
Optical parameters reflect presence of residual disease. 50x bicubic interpolated images of [β-carotene], <µ_s_′>, and [β-carotene]/<µ_s_′> from a A) negative and B) positive margin in 2 different patients with MBD-3. Sites with corresponding histopathology are highlighted with diagnoses of adipose (A), adipose plus fibroglandular (A+FG), and ductal carcinoma *in situ* (DCIS). C) eCDFs of the pixels from the representative images in panels A and B.

When areas of malignancy are present, the tissue composition landscape should shift to one with less fat and more fibroglandular components, i.e. one with less β-carotene and higher <µ_s_′>. Figure 5A shows [β-carotene], <µ_s_′>, and [β-carotene]/<µ_s_′> in a positive and negative margin from patients matched for breast density (MBD-3). In the positive margin there are lower values of [β-carotene] and [β-carotene]/<µ_s_′> and higher values of <µ_s_′>present as compared to the negative margin; in these particular images, these lower values of [β-carotene]/<µ_s_′> were histologically-confirmed to correspond to sites with ductal carcinoma *in situ* (DCIS) that was less than 0.5 mm below the margin surface. The benign sites, which had fattier composition, had higher [β-carotene] and [β-carotene]/<µ_s_′> values and lower <µ_s_′> values, although fibroglandular tissues obscure some of the differences between benign and malignant tissue. Figure 5C shows the eCDFs for each of these optical variables, comparing the negative and positive margin distributions. These eCDFs show the expected trend for increased <µ_s_′> in the positive margins compared to the negative margins. Interestingly, the [β-carotene] eCDF’s show that the positive margin is characterized by a greater proportion of very low [β-carotene] values (<25 µM) compared to the positive margin, but that it also contains a considerable fraction of pixels with [β-carotene] values higher than the negative margin. Even though these samples were matched for MBD, again this speaks to the inter-patient variability in tissue composition. However, the fact that the presence of malignant sites results in markedly low [β-carotene] values, along with a shift to higher <µ_s_′> values, increases the contrast in the [β-carotene]/<µ_s_′>parameter between positive and negative sites within the positive margin distribution. In other words, malignant sites with very low fat content and correspondingly low [β-carotene] values, would show better contrast if the surrounding fat had a very high [β-carotene] concentration.

**Figure 6:**
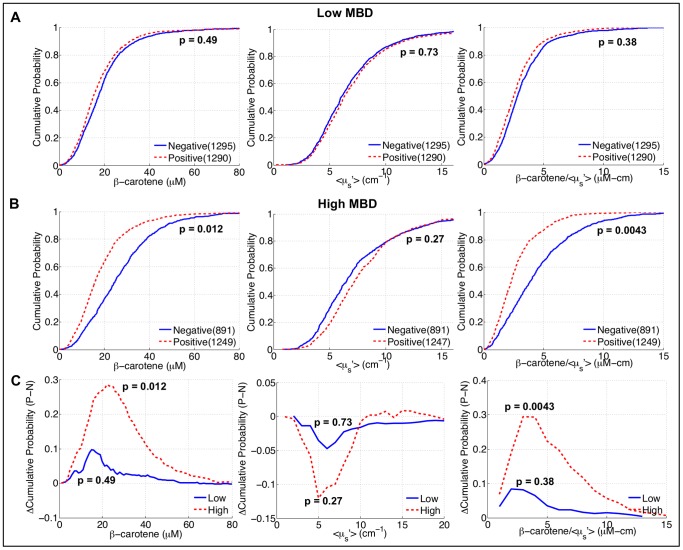
Optical contrast between negative and positive margins, stratified by mammographic breast density. A–B) eCDFs of all measured sites from negative and positive, neoadjuvant-naïve margins, separated by radiographic breast density. The *P*-values indicated correspond to modified Kolmogorov-Smirnov tests between: 1) negative versus positive margins in low density patients and 2) negative versus positive margins in high density patients. C) Difference eCDFs calculated between the positive and negative margin eCDFs of all measured sites for both low and high density breasts. Negative values indicate the negative margin distribution has higher values of the optical variable.

Figure 6 shows eCDFs of all measured image pixels from negative (2,186 measurements) and positive margins (2,539 measurements), stratified by breast density. These eCDFs show the trends that we expected for this shifting biological landscape: decreased [β-carotene], increased <µ_s_′>, and decreased [β-carotene]/<µ_s_′> in the positive margins compared to the negative margins. The significant increase in baseline [β-carotene] levels in the negative margins of high density patients, although originally unexpected, actually served to markedly improve contrast between positive and negative margins in this cohort of patients (this is also demonstrated in Figure 6C, where there is a large difference between the [β-carotene] values in benign and malignant sites in the positive margin). The ratio of [β-carotene] to <µ_s_′> also benefited from the differences in the negative margins due to breast density, and was found to be the most useful parameter in discriminating positive and negative margins. Figure 6C shows the difference in eCDFs (positive minus negative margin distributions) for low and high density patients and also shows the increased contrast in the high density patients.

A Conditional Inference Tree (CIT) model was fitted to the dataset using the 15 image descriptive variables described in the Methods. The CIT model can be thought of as a set of “rules” which are applied to any candidate margin image to determine whether it will be classified as positive or negative. For this model, the margin samples were first classified into low and high breast density subgroups (MBD ≤2 and MBD ≥3, respectively) prior to construction of the predictive model – this was in an effort to account for the differences in baseline optical variables between MBD subgroups previously observed. This in effect creates a single decision tree model, in which the first decision node is MBD subgroup. The primary quantitative parameter chosen in both the low and high MBD subgroups was the ratio of [β-carotene]/<µ_s_′>; however, the statistical parameter computed on that variable was different between the MBD subgroups. The other quantitative parameter selected by the CIT model was <µ_s_′>, and in particular, the Kolmogorov-Smirnov statistic, which compared a given margin sample <µ_s_′> distribution to the distribution of <µ_s_′> from all pixels from all positive margins. Table 2 summarizes the predictor variables selected by the conditional inference tree (CIT) model for each MBD-subgroup. Variable 1 in the Low MBD model is the percentage of [β-carotene]/<µ_s_′> image pixels below 1.2388 µM-cm, which reflects the proportion of the image with either very low [β-carotene] values or very high <µ_s_′> values, or both, which would be indicative of malignant regions (this is also demonstrated in Figure 5). Variable 2 in the Low MBD model is the 2-sided Kolmogorov-Smirnov statistic for <µ_s_′>. In this case, the Kolmogorov-Smirnov statistic for <µ_s_′>, which compares the <µ_s_′> eCDF of any given margin image to the <µ_s_′> eCDF of all pixels from all positive margins (the reference distribution), is helpful in further classifying these margin samples. Samples with a Kolmogorov-Smirnov statistic of 0 indicate that their pixel distribution was drawn from the reference distribution; therefore, samples with lower Kolmogorov-Smirnov statistics have distributions which more closely mimic the reference distribution of all positive margins, and are therefore more likely to be positive. For the high breast density subgroup, a single variable, the median of the [β-carotene]/<µ_s_′> margin distribution, was selected by the CIT model. In this case, positive margins were characterized by a lower median (0.5 quantile) [β-carotene]/<µ_s_′> than negative margins (as observed in Figure 5C). Taken together, these results indicate that the presence of residual carcinoma on the cancer margin indeed results in a shift in breast tumor margin morphology to one with less fat and more fibrous and glandular components, which is reflected as a shift to lower values of [β-carotene]/<µ_s_′>. In addition, the presence of residual carcinoma results in a <µ_s_′> image distribution, which is similar to the <µ_s_′> distribution for all pixels from positive margin samples.

**Table 2:** Summary of predictor variables selected by the conditional inference tree model, stratified by mammographic breast density (MBD).

Model	Optical Parameter(Unit)	Image Descriptive Parameter
***Low MBD***		
Variable 1	[β-carotene]/<µ_s_′>(µM-cm)	% image pixels <1.2388 µM-cm
Variable 2	<µ_s_′> (cm^−1^)	2-sided Kolmogorov-Smirnov statistic
***High MBD***		
Variable 1	[β-carotene]/<µ_s_′>(µM-cm)	Median value in image

Table 3 contains a summary of the performance of the technology for intra-operative detection of close or positive margin status. Margin assessment based on quantitative spectral imaging would have detected close/positive margins with 74% sensitivity (65% and 83% in low and high density breasts, respectively) and 86% specificity (92% and 76% in low and high density breasts, respectively). Overall, the surgeon sensitivity was 65% (69.6% and 60.9% in low and high density breasts, respectively), which was calculated based on the number of positive primary margins for which an additional intra-operative shaving was not obtained. However, determination of the true surgeon specificity is not straightforward, since at our institution some surgeons obtain additional shavings of multiple margins as a matter of course, whether or not they believed residual disease to be present – this had the effect of artificially increasing the false-positive rate (thereby decreasing the specificity), if we calculate it as the number of times the surgeon took a shaving of a negative primary margin. Therefore, although we compute the surgeon specificity (26%, Table 3) for comparison to the device, the preceding caveat applies. The surgeon sensitivity is also potentially decreased due to the inability, in some cases, of the surgeon to remove extra shavings of the anterior and posterior margins when they are abutting skin and muscle, respectively. This is demonstrated in Table 3 by the calculated sensitivity of 48% in anterior and posterior margins, compared to 86% in all other margin orientations. Therefore, the true surgeon sensitivity is likely more accurately reflected by the higher number, and is consistent with the eventual repeat surgery rates (27.1% of patients overall, or 21.1% (8/38) in low MBD patients and 34.4% (11/32) in high MBD patients). (Due to the difficulty in calculating the true surgeon performance on the primary specimen against histopathology, we do not report PPV, NPV, and overall accuracy.) 74% of the margins imaged in this study were in the MBD2 and MBD3 subgroups, which indicate that the lowest and highest density subgroups (MBD1 and MBD4) were unequally represented in this dataset. Although the sensitivity of the device and the surgeon are relatively similar overall, when broken down by MBD the sensitivity of the device is much higher in the high density subgroup.

**Table 3:** Sensitivity (Se), specificity (Sp), positive predictive value (PPV), negative predictive value (NPV), and classification accuracy (A) of the device and the surgeon.

Samples (n)	Se (%)	Sp (%)	PPV (%)	NPV (%)	A (%)
***Device Performance***					
All (88)	74	86	85	75	80
*MBD 1–2 (48)*	65	92	88	74	79
*MBD 3–4 (40)*	83	76	83	76	80
*Anterior & Posterior (40)*	64	73	80	55	68
*Other orientation (48)*	86	93	90	89	90
Surgeon Performance*					
All (88)	65	21			
*MBD 1–2 (48)*	70	28			
*MBD 3–4 (40)*	61	12			
*Anterior & Posterior (40)*	48	33			
*Other orientation (48)*	86	15			

Performance within each MBD subgroup is given. *The surgeon’s performance is based on the primary specimen (and no additional shavings) taken during the first operation.

Table 4 provides a breakdown of the false negatives and false positives for the CIT model and the surgeons’ performance. These numbers are broken down by the type of cancer found at the margin, the surgical margin status, and MBD. Of the 12 false-negatives in the CIT model, close margins and margins containing DCIS had higher percentages (75% and 33%, respectively) of being missed than positive margins or margins that contained IDC (25% and 25%, respectively). This was also true of the surgeons’ performance where 38% DCIS margins were missed and 63% close margins were missed. Four out of the 6 false positives in the model were associated with histologic features that may explain why they were incorrectly diagnosed. One of the measured false-positive margins consisted of fibrocystic change and fibroadenoma; two of the measured margins had adjacent margins that contained atypical ductal hyperplasia and fibrocystic change; and another measured margin had cancer ∼4 mm from the surface.

**Table 4:** Number of false negative (FN) and false positive (FP) margins (stratified by margin histology and surgical margin status) and patients (stratified by MBD) calculated from the surgeon performance, as well as, the performance of the device.

		# of FN (%)		# of FP (%)	
		Surgeon	Device	Surgeon	Device
**Margin Histology**	**IDC**	3 (19)%	3 (25%)	0 (0%)	0 (0%)
	**DCIS**	6 (38%)	4 (33%)	0 (0%)	0 (0%)
	**IDC/DCIS**	1 (6%)	1 (8%)	0 (0%)	0 (0%)
	**Other**	6 (38%)	4 (33%)	0 (0%)	0 (0%)
	**No Tumor**	0 (0%)	0 (0%)	33 (100%)	6 (100%)
**Surgical Margin Status**	**Negative**	0 (0%)	0 (0%)	33 (100%)	6 (100%)
	**Close**	10 (63%)	9 (75%)	0 (0%)	0 (0%)
	**Positive**	6 (38%)	3 (25%)	0 (0%)	0 (0%)
**MBD**	**1**	2 (13%)	3 (25%)	4 (18%)	1 (20%)
	**2**	5 (31%)	5 (42%)	9 (41%)	1 (20%)
	**3**	8 (50%)	3 (25%)	7 (32%)	2 (40%)
	**4**	1 (6%)	1 (8%)	2 (9%)	1 (20%)
**Measured Margin**	**Anterior and Posterior**	13 (81%)	9 (75%)	10 (30%)	4 (67%)
	**All Others**	3 (19%)	3 (25%)	23 (70%)	2 (33%)

### Summary and Conclusions

The technology presented here seeks to bridge the gap between the requirement for microscopic resolution and cm^2^ imaging areas and millimeter sensing depth for tumor margin assessment, by leveraging quantitative optical spectral imaging to report on the underlying composition of the interrogated tissue. Specifically, the device has an imaging area throughput of 5 cm^2^/min with 5 mm lateral resolution and 0.5–2 mm sensing depth; higher lateral resolution can be achieved by finer sampling at the expense of imaging throughput. An important advantage of this technique is the ability to be sensitive to the tissue down to 2 mm below the tissue surface, which is important for detection of residual cancer lying surreptitiously below the surface. A point-scanning probe based on RF spectroscopy [35]–[37], the MarginProbe, commercialized by Dune Medical, has recently obtained FDA approval for breast tumor margin assessment. Compared to that technology, notable differences of our device are the use of optical wavelengths, and the ability to quickly collect images or maps of the surface. Other emerging optical technologies meant to address this problem include point-scanning reflectance spectroscopy [38]–[41], spatial frequency domain imaging [38], and optical coherence tomography [42]. The point-scanning reflectance techniques are fundamentally equivalent to our approach, and nicely demonstrate what is possible in terms of spatial resolution with the technique when the specimens are densely sampled (similar resolutions could be achieved with our multi-point-scanning system by translating the probe in finer increments). Spatial frequency domain imaging is a relatively new wide-field reflectance imaging technique, which is capable of acquiring quantitative optical property maps of the tissue, similar to our system, and was shown to be sensitive to absorbing and scattering inclusions beneath the surface of cleverly-designed contrast detail phantoms [38]. The strengths of our reflectance approach compared to alternate implementations are the use of parallel-pixel detection (8 parallel pixels in the system used for this study, 49 parallel pixels in the 2^nd^-generation system), the robust and simple calibration scheme (requiring a single measurement of an off-the-shelf Spectralon reflectance standard for each imaging session), and the contact sensing geometry which, coupled with the specimen holder, allows well-defined sampling characteristics and readily facilitates accurate pathologic co-registration. Optical coherence tomography is a depth-resolved technique which observes information related to light scattering of tissue from suitable depths, but at this time has not been scaled for observation of >1 cm^2^ fields of view [42], [43], although the potential for such scaling exists. In addition, any microscopic imaging approach will result in a preponderance of image data (i.e., more data than a human can reasonably assess in an intra-operative timeframe), which must be analyzed with an appropriate computational algorithm, which can detect spatial signatures associated with residual cancer.

The technique presented here (along with other diffuse reflectance techniques), although not capable of microscopic image resolution, does offer a pragmatic solution that has potential as a clinically translatable tool for tumor margin assessment. Resolution specifically pertains to the ability to spatially separate the locations of adjacent structures, whereas sensitivity pertains to the ability to quantify a measureable change in contrast. Rather than leverage high spatial *resolution*, we instead leverage *sensitivity* to optical sources of contrast, which are related to tissue composition. In other words, sensitivity to small tumor foci would be more related to the difference in optical properties between benign and malignant tissue, than to the spatial resolution of the device (as demonstrated in the study by Laughney [38]). A previous study by our group using a single channel version of the device determined that the technique could detect malignancy with 78% sensitivity when it comprised <25% of the interrogated volume; that sensitivity went up to 84% when malignancy comprised 25–50% of the interrogated volume [44]. Those results reinforce our findings that the sensitivity to “close” margins (where the malignancy is below the surface) is less than that for “positive” margins (where the malignancy is at the surface and comprises more of the sensing volume). However, as shown in Table 4, our device would have resulted in fewer false negatives than the surgeon for both close and positive categories, respectively.

The primary sources of contrast in this study were β-carotene concentration, the reduced light scattering coefficient, and the ratio of β-carotene concentration to the scattering coefficient. These parameters are sensitive to a shift in the tissue composition landscape from a “normal” mix of adipose, fibroadipose, and fibroglandular tissues, to a landscape with decreased adipose and an increased fibroglandular component (associated with malignancy). We did find differences in the baseline β-carotene concentration in the adipose tissues of breasts with differing mammographic breast density, which required these cohorts of patients to be considered separately. However, we do not envision this as a fundamental limitation of our approach, since mammographic breast density is generally known prior to surgery and could be used as *a priori* information in the predictive model.

The differences in adipocyte size and [β-carotene] with breast density were an unexpected finding, but actually served to increase optical contrast in high density breasts. This was a fortuitous finding given that surgeons have less flexibility to resect large volumes in high density breasts (which tend to be smaller), therefore increasing the need for an intra-operative assessment tool. From this cohort of 70 patients the percentage of patients who returned for a second surgery was 21.1% (8/38) of low density patients and 34.4% (11/32) of high density patients. This higher re-excision rate in high-density patients is consistent with others reported in the literature. For example, Bani et al [20] found that higher MBD was associated with higher re-excision rates; specifically their re-excision rates were: 18% (MBD-1), 18% (MBD-2), 22% (MBD-3), and 42% (MBD-4). These studies speak to the fact that surgeons face greater difficulties in excising tumors in a denser breast and that an optical device would especially benefit this patient population. However, in our study, the number of combined close and positive margins on the primary specimen was identical in both MBD cohorts, which points to the need for an intra-operative assessment tool even in cases where the surgeon is free to resect large tissue volumes. With regard to the relationships observed between adipocyte size and β-carotene concentration, it is not clear if it is simply the result of higher concentrations due to smaller individual storage volumes in the smaller adipocytes, or if β-carotene plays a more active role in the determination of adipocyte size. A limitation of this analysis was the small sample size and the assumption of independence in the statistical tests used. However, for the purposes of our study, observing these trends helped us to account for some of the inter-patient variance and improve the diagnostic potential of the device, and suggest an area of further study.

There are a number of limitations of the study that must be addressed. One limiting factor in this study was the lack of a control group consisting of entirely normal breast tissue (i.e. reduction mammoplasty specimens). Analysis of normal tissue will be essential to ensure that the relationship between mammographic breast density and adipocyte size does exist independent of malignancy and is not due to a cancer “field effect”. Future studies will include non-cancerous breasts to validate the observation that the β-carotene concentration and adipose size are related and modulated by breast density, independent of the presence of malignancy. Another limitation of this particular study was the imaging of only 1–5 full margins per specimen, due to time constraints. The bottle-neck in the process was the need to manually translate the probe to cover the entire margin. In this study, the entire process (acquiring the sample, mounting in the holder, imaging, book-keeping, data analysis, and clean-up) took between 20–25 minutes, or roughly the amount of time between acquiring the sample and the patient leaving the operating room. The number of margins, which were imaged for each patient was thus limited by the size of the sample and the length of time available in the operating room. Imaging of all sides of a 100 cm^2^ specimen would take approximately 20 minutes with the 8-channel probe described here, in terms of probe translation and imaging/analysis time (5 cm^2^/min area throughput, limited by manual probe translation time). We note however, that this process has been sped up through the incorporation of approximately 6 times as many channels in the imaging probe to yield a 49-channel probe in a commercial prototype (Zenascope PC49, Zenalux Biomedical, Inc., Durham, NC). The entire collection, processing, and display process for a full margin has been automated and can be completed in 30 seconds with the click of a single button in the software, demonstrating the feasibility of scaling this technology for clinical translation. With this 49-channel version, a 100 cm^2^ BCS specimen can be scanned in ∼3 minutes at 5 mm lateral resolution (imaging area throughput = 35 cm^2^/min). With regards to our findings related to β-carotene concentration in adipose tissues, in this study, we did not collect the diet or supplement history of the patients, so we do not know how dietary β-carotene intake affected the measured adipose β-carotene concentration levels. In fact, we do not know of a study that has systematically investigated to what extent excess β-carotene in the diet is stored in the adipose tissue. However, we did not observe a clear correlation between breast density and BMI in our study, which indicates that our observations of differences in adipose β-carotene concentration across mammographic density levels was not biased by BMI (assuming that patients with higher BMI might have poorer nutrition and lower β-carotene intake). However, this is an open area for investigation and must be considered in future studies with the device. A final limitation of the work presented here is that the CIT predictive model has not been validated on an independent dataset, and is therefore an estimate of the model prediction accuracy only; validation studies are underway.

In conclusion, quantitative optical spectral imaging may provide a practical adjunct to established clinical paradigms in breast conservation surgery and histopathology, by providing a rapid survey of the margin surface that is reflective of underlying tissue composition. In particular, the technology could be especially useful in patients with dense breast tissue, which present a greater challenge to the surgeon in obtaining negative margins. The study described here was not designed to definitively demonstrate the advantage of this technique over the unaided surgeon with respect to patient outcomes, but it does demonstrate in a relatively large number of specimens the potential clinical utility and translational feasibility of such a device to detect residual cancer on the primary specimen as compared to histopathology. A 150-patient prospective validation study, using a faster second-generation version of the device, is currently underway at Duke University Medical Center.
